# Socio-economic inequality in malnutrition among children in India: an analysis of 640 districts from National Family Health Survey (2015–16)

**DOI:** 10.1186/s12939-019-1093-0

**Published:** 2019-12-27

**Authors:** Shrikant Singh, Swati Srivastava, Ashish Kumar Upadhyay

**Affiliations:** 1 Department of Mathematical Demography and Statistics, International Institute for Population Sciences, Govandi Station Road, Deonar, Mumbai, 400088 India; 2 International Institute of Population Sciences, Govandi Station Road, Deonar, Mumbai, 400088 India

**Keywords:** Malnutrition, Stunting, Underweight, Socio-economic inequality, Wagstaff decomposition

## Abstract

**Background:**

Despite a fast-growing economy and the largest anti-malnutrition programme, India has the world’s worst level of child malnutrition. Despite India’s 50% increase in GDP since 1991, more than one third of the world’s malnourished children live in India. Among these, half of the children under age 3 years are underweight and a third of wealthiest children are over-nutrient. One of the major causes for malnutrition in India is economic inequality. Therefore, using the data from the fourth round of National Family Health Survey (2015–16), present study aims to examine the socio-economic inequality in childhood malnutrition across 640 districts of India.

**Method:**

Concentration curve and generalized concentration index were used to examine the socioeconomic inequalities in malnutrition. However, regression-based decomposition methodology was used to decomposes the causes of inequality in childhood malnutrition.

**Result:**

Result shows that about 38% children in India were stunted and 35% were underweight during 2015–16. Prevalence of stunting and underweight children varies considerably across Indian districts (13 to 65% and 7 to 67% respectively). Districts having the higher share of undernourished children is coming from the particular regions like central, east and west part of the country. On an average about 35% of household in a district having the access of safe drinking water and 42% of household in a district exposed to open defecation. The study found the inverse relationship between district’s economic development with childhood stunting and underweight. The concentration of stunted as well as underweight children were found in least developed districts of India. Decomposition approach found that practice of open defecation is positively influenced the inequality in stunting and underweight. Further, inequality in undernutrition is accelerated by the height and education of the mother, and availability of safe drinking water in a district.

**Conclusions:**

The districts that lied out in a spectrum of developmental diversity are required some specific set of information’s that covering socio-economic, demographic and health-related quality of life of people in those backward districts. More generally, policies to avail improved water and sanitation facility to public and female literacy should be continued. It is also important to see that the benefits of both infrastructure and more general economic development are spread more evenly across districts.

## Background

Since the last few decades, childhood malnutrition became one of the major public health concerns in low and middle-income countries. Estimates from the United Nations Children’s Fund (Unicef) suggest that, globally about 165 million children under the age of 5 years were found to be stunted (low height for age), 101 million children were found to be underweight (low weight for age), and 52 million children were found to be wasted (weight for height) [1]. Further, from the estimates from United Nation (UN), about 6.3 million under age-five mortality were occurred in India, of which 45% died due to malnutrition [2].

India alone accounts for more than 61 million stunted children (low height for age), 47 million underweight children (low weight for age) and 25 million wasted children (weight for height). Estimates from the National Family and Health Survey (2015–16) shows that in India, about 38% of the children under the age of five year are stunted (low height for age), 36% of the children are underweight (low weight for age), and 18% children are wasted (weight for height) [3].

Horton (1999) reported that malnutrition is a major concern of development and associated with enormous human and economic causes [4]. Poor health of the children erodes social and economic gains, put countries in a vicious cycle of poor nutritional status, high disease burden and increases poverty. A large number of studies had reported the short-term and long-term impact of early childhood malnutrition in developing countries [5–11]. The short-term effects include weaker immune system, a higher risk of developing diarrheal disease, acute respiratory infection, and delay in motor skills and cognitive and social development during childhood [5–8]. The long-term effects include high blood pressure, obesity, diabetes, and heart disease during adulthood.

A number of previous studies had examined the socio-economic gradient of child malnutrition in India, some of the study reported that childhood malnutrition was mainly concentrated among households with low socio-economic status [12–21]. Majority of these studies were examined the inequalities in childhood malnutrition on the basis of household economic status. Moreover, measuring health inequality on the basis of household economic status may pose limitations in terms of identifying and reaching disadvantaged subpopulations, as the poorest segment of the population may be located throughout different regions of a country [15, 17, 22–28].

India’s administrative division of 29 states and 7 union territories falls into 640 districts. Therefore, variations in basic demographic and health indicators are prevalent not only in the states but also across the districts. Recent estimates from NFHS-4 (2015–16) suggest that childhood malnutrition varies considerably across Indian districts. The prevalence of stunting among children under age five was lowest in Kollam (13%), a district of Kerala and highest in Bahraich (65%), a district of Uttar Pradesh. Similarly, the prevalence of underweight was lowest in Aizwal (7%), a district of Mizoram and highest in Pashchimi Singhbhum (67%), a district of Jharkhand. Therefore, due to the extensive heterogeneity in childhood malnutrition across Indian districts, the present study aims to determine the socio-economic inequality in childhood malnutrition (stunting and underweight) across districts of India, by taking district as a unit of analysis. The key advantage of doing district level analysis is helpful to give focus on decentralized planning, as the recent interventions to reduce inequities are likely to be implemented at the local administrative level. This may be a useful tool for benchmarking, with implications for resource allocation, planning and evaluation, as the districts are the smallest level of unit to monitor the majority of the ongoing child health intervention program in India. The analysis of the variations across districts is also necessary for development of district level program and interventions in the context of developmental vulnerability and identification of high priority districts to strategized intersectoral co-ordinations involving various impartments like health, social welfare, women and child development. Further, the importance of focusing on district level analysis rather than household level analysis is important, as district-level policies undertaken and implemented by district-level politicians and officials may often be of critical importance in determining these outcomes. Also, the influence of social norms which have been undergoing rapid changes in India in recent years on child mortality, fertility, etc., is probably more adequately captured in district-level than in household-level analysis. Therefore, it has been observed that the district and household-level analyses are not, contradictory even it has been viewed as being complimentary.

Given the lack of study on the topic, using latest round of data from fourth round of National Family Health Survey conducted in 2015–16, the present study intended to examine the socioeconomic inequalities in childhood malnutrition in India. Further, study also examined the causes of socioeconomic inequalities in childhood malnutrition in India.

## Data and methodology

### Survey data

The study used data from the fourth round of National Family Health Survey (NFHS-4) conducted during 2015–16 in all states and union territories of India. The NFHSs is a large-scale, cross-sectional, multi-round survey which is nationally representative sample of households throughout India. Till date, four rounds of the NFHS has been conducted in India during 1992–93, 1998–99, 2005–06 and 2015–16 under the stewardship of the Ministry of Health and Family Welfare, Government of India.

The survey provides state and national level estimates of demographic and health parameters as well as data on various socioeconomic and program dimensions, which are critical for implementing the desired changes in demographic and health parameters. NFHS adopted two-stage sample design in rural areas, and three-stage sample deign in urban areas. In the rural area, Primary Sampling Units (PSUs) (villages) used to select at the first stage, followed by the selection of household at the second stage. In urban areas, a three-stage procedure was followed. In the first stage, wards were selected with PPS sampling. In the next stage, one census enumeration block (CEB) was randomly selected from each sample ward. In the final stage, households were randomly selected within each selected CEB. The response rates of the survey were about 90% to 98%, and there were only small variations in these rates among different states of the country [3]. The total sample size of the children under age 5 years preceding the survey was 224,449. The present study is based on the children born within 5 years preceding the survey for which complete information on height, weight and other indicators are available. Therefore, after deleting about 40,000 flagged observations (with unusual measures and incomplete/missing information), the final analysis was carried out on 184,567 children born within the 5 years preceding the survey.

### Variables description

The outcome variable of interest are ‘childhood stunting (low height for age)’ and ‘childhood underweight (low weight for age)’ which is the chronic nutritional status of children. Initially ‘childhood stunting’ and ‘childhood underweight’ was coded in dichotomous (0 ‘not-stunted/underweight’, 1’stunted/underweight’). Further, the study makes outcome variable at the representative at district level, percent stunting and percent underweight were taken as final outcome variable.

Apart from these a number of variables has been taken as the exposure variables in the study. The detailed description of these variables are given in Table 1.

**Table 1:** Variable definitions and sample means or percent (standard deviations) with minimum and maximum value in India, 2015–16

Variables	Definitions	Mean or %	Std. Dev.	Min	Max
Stunting	District’s with mean stunted children below age 5 years	38.36	9.69	13.01	65.11
Underweight	District’s with mean underweight children below age 5 years	34.50	11.58	7.00	66.95
Female Child	Percent of female child in a district	48.28	2.70	36.12	60.25
Birth size (> = normal)	Percent of women reported to have birth size average and above in a district	88.07	5.17	70.55	98.67
Birth order	District’s average birth order	2.27	0.42	1.37	3.60
Unwanted birth	Percent of unwanted birth in a district	4.17	2.96	0.00	16.08
Height of mother	Average height of the women (aged 15–49) in a district	152.78	2.94	147.75	170.34
Female literacy	Mean year of schooling of women (aged 15–49) in districts	6.22	2.21	1.45	13.25
Scheduled caste and tribes	Percent of district’s female population belonging to scheduled caste and tribes	40.55	23.88	1.31	100.00
Muslim	Percent of district’s female population belonging to Muslim household	15.79	19.57	0.00	100.00
Safe drinking facility	Percent of household in a district with access of safe drinking water	35.35	28.05	0.15	98.92
Open defecation	Percent of household in a district exposed to open defecate	42.10	27.43	0.00	89.12
Rural Population	Percent of district’s female population living in rural area	76.23	19.12	0.00	100.00
North	Consist of the states-Haryana, Himanchal Pradesh, Jammu & Kashmir, Punjab, Rajasthan, Chandigarh and Uttarakhand	0.19	0.39	0.00	1.00
Central	Consist of the states- Chhattisgarh, Delhi, Madhya Pradesh and Uttar Pradesh	0.29	0.46	0.00	1.00
East	Consist of the states- Bihar, Jharkhand, Odisha and West Bengal	0.21	0.41	0.00	1.00
Northeast	Consist the states-Arunanchal Pradesh, Assam, Manipur, Meghalaya, Mizoram, Nagaland, Sikkim and Tripura	0.14	0.35	0.00	1.00
West	Consist of the states- Goa, Gujrat, Maharashtra, Daman & Diu, Dadar & Nager Haveli A& N Island	0.07	0.25	0.00	1.00
South	Consist of the states-Andhra Pradesh, Karnataka, Kerala, Lakshadweep, Puducherry, Tamil Nadu and Telangana	0.10	0.30	0.00	1.00

## Methodology

### Socio-economic status

According to the aim of the study, present study determined the socio-economic inequality in childhood malnutrition by using concentration index. The measurement of socio-economic inequality required a variable which allows us to rank the districts according to their level of economic development. The ideal variable to measure district’s economic development would be per capita district income. But, in India there are no data source is available which can provide direct information of district level income. Due to unavailability of district’s direct income, present study took socio-economic status of household’s as the proxy of economic development of district. The study calculated the socio-economic status in such a way that it could represent a district’s economic development (as a proxy). The process of the construction of socio-economic status is described below.

### Construction of socio-economic status

Measuring socioeconomic status are always an issue of debate. As direct information of income and expenditures are not available in NFHS data and circumstances where available are subject to serious bias. The present study uses principal component analysis to measure the socioeconomic status [29]. The study used consumer durables, housing quality, services and education of head of households to construct socioeconomic status (SES). Present study did not include the information on the type of toilet and source of drinking water, while developing the SES, because both of the variables are considered to have a direct relation to children’s nutritional status. The recalculated SES was used as a suitable proxy for socio-economic status, as mentioned in the literature [30].

### Measure of socio-economic inequality

The present study used the concentration index to access the socio-economic inequality in childhood undernutrition in India. The concentration index can be defined simply as twice the covariance between the health variable (y: let childhood stunting) of individual i and the ranking of the socioeconomic status, r, divided by the mean of the health variable (μ):

1 $$ \mathrm{CI}=\frac{2}{\upmu}\operatorname{cov}\left({\mathrm{y}}_{\mathrm{i}},{\mathrm{r}}_{\mathrm{i}}\right) $$

This is the widely used measure because it ranks the individual across SES, sensitive to changes in population distribution across SES and they can assess relative and absolute socio-economic inequality [20, 43].

### Decomposition of concentration index

Even though concentration indices are relevant to shows the extent of socioeconomic-related inequalities in childhood malnutrition, but it cannot explain the factors that contributed to observed inequalities. Therefore, the present study has great interest to know these factors, as they are crucial for policy and for addressing some of the underlying ‘causes’ of inequality.

To address this concern, present study used the regression-based-decomposition methodology to decomposes the concentration index explain the inequality in childhood malnutrition. The contribution of each factor to socio-economic related inequality in childhood stunting/underweight is computed as the product of the sensitivity of child stunting/underweight concerning each factor and the degree of socioeconomic-related inequality in each factor. As the outcome variable of this study is continuous in nature therefore study used linear probability model to decompose the concentration index. Thus, the expression of model is- -

$$ {y}_i=\alpha +\sum \limits_j{\beta}_j{x}_{ji}+{u}_i $$

Here *β*_*j*_ is the probability of malnourishment (stunted/underweight), associated with j determinants [33]. The *CI*_*y*_ can be decomposed as follow-

$$ {CI}_y=\sum \limits_j\left(\raisebox{1ex}{${\beta}_j\overline{x_j}$}\!\left/ \!\raisebox{-1ex}{${\mu}_y$}\right.\right){CI}_{x_j}+\frac{CI_u}{\mu_y} $$

where the second term on the right-hand side represents income related socio-economic inequality in outcome variable that is not explained by systematic variation in x’s by income. But we are interested in the first term on the right-hand side of the equation, which represents the contribution of each determinant to CI(y). All the analysis was carried out in STATA 13 version.

## Results

Table 1 shows the descriptive statistics of the variables taken in the study. After deleting missing and flagged observation from the data, study observed the mean stunted children in India is about 38% and mean underweight children in India is 35%. On an average, about 48% of household in a district have a female child. More than three-fourth(88%) of women in a district were delivered their babies with normal birth size. Present study further found that the district’s average birth order is 2.3, however on an average about 4% of births in a district was unwanted. Women’s average height in a district is about 153 cm, however average female literacy is about 6 years. About 41% of district’s female population belongings to scheduled caste/tribes and 16% population belongs to Muslim household. On an average about 35% of household in a district having the access of safe drinking water and 42% of household in a district exposed to open defecation. Study also shows that about 76% of district’s child population residing in rural areas.

### Measurement of socio-economic inequality

Table 2 shows the distribution of stunting and underweight among children below age 5 years, where districts were ranked according to district’s economic development. The study found the inverse relationship between district’s economic development with childhood stunting and underweight. This inverse association between economic status and childhood malnutrition indicates that economically developed districts have lower prevalence of childhood malnutrition than less developed districts. For instance, among least developed districts there are about 48% of stunted and 44% of underweight children however, in most developed districts the prevalence of stunting and underweight was about 29% and 26% respectively.

**Table 2:** Distribution of average stunting and underweight among children below age 5 years, by district’s economic development level in India

Quintile	Stunting			Underweight		
	Mean (std dev)	min	max	Mean (std dev)	min	max
1 (least developed)	48.2 (0.0)	48.2	48.3	44.4 (0.0)	44.3	44.4
2	42.0(0.1)	41.9	42.1	38.4(0.1)	38.3	38.5
3	39.2(0.1)	39.1	39.4	37.2(0.1)	37.1	37.4
4	36.2(0.0)	36.1	36.3	34.5(0.1)	34.4	34.6
5 (most developed)	28.5(0.0)	28.4	28.5	26.2(0.0)	26.1	26.3

**Fig. 1:**
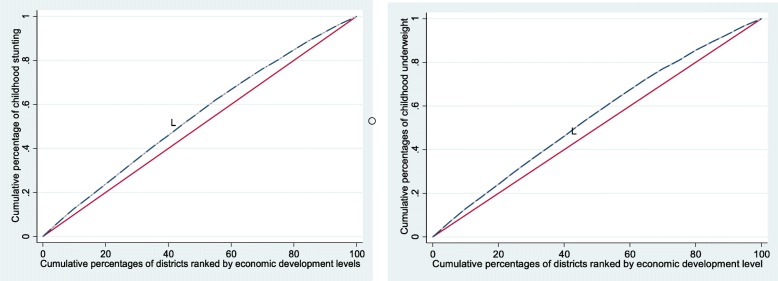
Concentration curve of stunting and underweight

The results of concentration index to examine the socioeconomic inequalities in childhood stunting and underweight are presented in Table 3. Result shows that about 10% of the socio-economic inequality in childhood stunting (CI = − 0.100) and 9% socio-economic inequality in childhood underweight (CI = − 0.098) across the districts of India. Negative value of CI indicates that the inequality is more in favors of the less developed districts (i.e., malnutrition is more concentrated in less developed districts) for both indicators. Concentration curve presented in Fig. 1 also confirms that stunting and underweight is typically higher amongst the less developed districts of India (as line L lies above the diagonal for both stunting and underweight).

**Table 3:** Decomposition of concentration Index for stunting and underweight among children below age 5 years in India,2015–16

Variables	Stunting				Underweight			
	Coefficient	Elasticity	C.I	% Contribution	Coefficient	Elasticity	C.I	% Contribution
Female child	−0.033	−0.042	−0.005	−0.190	−0.057	−10.493	−0.451	−0.481
Birth size (> = normal)	0.022	0.050	0.008	−0.404	0.094^*^	28.806	0.803	−2.348
Birth order	6.998^*^	0.391	−0.074	28.785	−2.731^*^	−12.044	−7.356	−8.997
Female literacy	−0.123^*^	−0.218	0.139	30.283	−0.215^*^	−0.433	0.139	61.170
Height of mother	−1.854^*^	−7.348	0.005	35.322	−1.680^*^	−5.773	0.481	28.182
Safe drinking facility	−0.007	−0.007	0.354	2.613	−0.041^*^	−0.982	0.353	3.530
Open defecation	0.093^*^	0.094	−0.259	24.275	0.141^*^	14.867	−0.259	39.178
Rural Population	−0.008	−0.015	− 0.132	−1.994	−0.033^*^	−5.971	− 0.132	−8.006
Muslim	−0.001	0.000	−0.120	− 0.046	−0.034^*^	0.074	−0.259	0.019
Scheduled caste and tribes	−0.036^*^	−0.036	− 0.468	−16.885	0.004	4.418	−0.599	2.690
Central	−0.886	−0.007	− 0.175	−1.187	0.771	1.038	−0.175	1.850
East	−4.833^*^	−0.027	−0.547	−14.509	−3.283^*^	−1.546	− 0.546	−8.587
Northeast	−4.660^*^	−0.017	− 0.286	−4.952	−9.274^*^	−0.479	− 0.285	−1.392
West	2.079^*^	0.004	0.413	−1.530	6.418^*^	2.330	0.413	−9.773
South	−1.904^*^	−0.005	0.500	2.405	−1.461	−0.493	0.499	2.503
Residual				18.020				0.460
Total			−0.101	100			−0.098	100
Adjusted R^2^	0.740				0.691			

*Significance at 5%

### Regression based decomposition (RBD)

The result of decomposition analysis has been given in Table 3 along with OLS regression result. OLS results are important in terms of identifying the variables which are statistically significant in the decomposition analysis. It has been noted that, if the coefficient estimate of the independent variables is statistically significant in regression result, then the contribution of that variables to explain socio-economic inequality will also be statistically significant in decomposition.

While observing variables related to mother characteristic, study found that the coefficient of birth order, height of the mother and female literacy is significant for both outcome variables, where height of the mother has been taken as the proxy of maternal nutrition. In fact, the sign of the coefficient of mother’s height and education are negative for both the indicators (stunting and underweight), which shows that district having higher proportion of taller and educated mothers have lower proportion of stunted and underweight children. It must be highlighted that the largest contributors to socio-economic inequality in childhood stunting and underweight are also comes from the height of mother (35 and 28% respectively) and female literacy (30 and 61% respectively).

Further, the coefficient of birth order is positively associated with childhood stunting (6.99, *p* < 0.05) but negatively associated with childhood underweight (− 2.73 *p* < 0.05). Also, the birth order of child has positive elasticity (0.391) with significant contribution to increase childhood stunting (29%) but have negative elasticity (− 12.04) with significant contribution to reduce underweight (8%). This portrays that districts having higher birth order had higher share of stunted and lower share of underweight children. Of the other variables, the coefficient of sex of the child are negatively associated with stunting (− 0.033, *p* > 0.005) and underweight (− 0.057, *p* > 0.005), however, the relationship is not statistically significant. Study did not find any significant effect of birth size on childhood stunting and underweight.

Moreover, improved source of drinking water and practice of open defecation was also found to be associated with childhood stunting and underweight in the study. Finding indicates a negative association of safe drinking water facility and rural population with malnutrition. Which means that district having more access of improved water facility have lower proportion of stunted (− 0.007, *p* > 0.05) and underweight (− 0.041, *p* < 0.05) children. Decomposition result indicates that improved source of drinking water has 3% contribution to generate socio-economic inequality in childhood stunting and about 4% contribution in socio-economic inequality in underweight. Additionally, the districts having higher proportion of rural population had lower proportion of stunted (− 0.008, *p* > 0.05) and underweight children (− 0.033, *p* < 0.05) in India. Likewise, analysis shows that the prevalence of stunting and underweight was higher among the districts having higher percent of practice of open defecation. Practice of open defecation was also having strongest positive contribution to produce inequalities in childhood stunting (24%) and underweight (39%) in India.

Also, districts having higher percent of scheduled caste/tribes was less likely to be stunted (− 0.036, *p* < 0.05) and underweight (− 0.004, *p* > 0.05) children. This finding reflects that districts having higher share of scheduled caste/tribe’s population having lower proportion of stunted and underweight children. However, this relationship is not statistically significant for underweight children. Further the study found that districts having higher share of Muslim population are negatively associated with stunting (− 0.001, *p* > 0.05) and underweight (− 0.034, *p* < 0.05) children.

So far regional effects are concerned, some of the regional dummies are showing positive association while some of them showing negative association with both outcome variables, which confirms the variation in proportion of stunting and underweight across regions compared to controlled region. In the survey year, central, northeast, east and southern region are less likely to be have stunted children compare to control region (i.e., north region), however only east, northeast and south region are less likely to be have underweight children compare to control region. Overall, the socioeconomic determinants included in our model explain 82% socio-economic inequality in childhood stunting and 99% inequality in childhood underweight. By inference, this means that the residuals or inequality in stunting explained by other factors is 18% and inequality in underweight explains by other factors is 0.5%.

## Discussion

In India, high level of undernutrition is much debated issues since the last two-three decades. According to Global Hunger Index report, India stood at 100th position among the 119th countries, which indicates the higher prevalence of under-nutrition and hunger related problem in country [34]. Using data from the fourth round of NFHS, the present study examined the socioeconomic inequalities in childhood stunting and underweight across 640 Indian districts. The study also made an effort to decompose the causes of the socio-economic inequality in childhood malnutrition. Disaggregating analysis at the districts level helps to unearths the disparities in nutritional outcomes among the children in India. Finding of the present study shows the inverse relationship between district’s economic development with nutritional outcomes and also highlighted that the poor nutritional status of children was more concentrated in less developed districts of India. A similar finding was also reported by study from India along with other child health related indicators [35–37]. However, the majority of the existing literatures examined the socioeconomic inequality at household level. A study by Bhattacharya have examined the socioeconomic inequalities in under-five mortality at district level in India and reported that socio-economic inequality in under-five mortality was more concentrated in least developed district of India. To the best of our knowledge, this is the first study in India which examined the socioeconomic inequalities in child malnutrition across Indian districts using latest round of data from NFHS.

Results of decomposition analysis suggest that mother's literacy and height had profound contribution to make equal distribution in nutritional outcome. This finding is consistent with previous studies conducted in other developing countries [22]. Possible explanation for this would appear to be the following. First, the role of female literacy becomes more significant when a developing country experiences a major spurt in the growth rate of the economy and the associated changes in society. The increased availability of health and other public infrastructures supporting maternal and child health with rapid economic development helps women to improve the health of women and their children, and the literate women are better able to take advantage of these opportunities. Second, in a bright economy, with more income opportunities, literate women are able to improve their incomes relative to those who are illiterate and so, are able to provide better health care for their children.

The decomposition analysis portrays that more equitable distribution of improved source of drinking water and toilet facility across all districts contributed to reduce childhood stunting and underweight in India. This association is also confirmed by findings of other studies which were carried out in other part of the world [38–41]. One possible reason to explain this association might be that children are more prone to be affected by environmental contamination as they start crawling, walking, exploring and putting objects in their mouths, which may increase the risk of ingesting faecal bacteria from both human and animal sources. This leads to repeated episode of diarrhea and intestinal worms, which in turn deteriorates the nutritional status of children [42]. Another possible reason to explain this association could be linked with the mother/household characteristic. There are many studies explored the association between the mother’s/ caregiver’s personal hygiene practices with childhood malnutrition [43, 44]. It is very clear that the efforts to maintain personal hygiene practices of both mother/household and child level will prevent children from diarrhea and other infectious diseases, which in turn contribute to reduce malnutrition among children.

Furthermore, study also found that a negative association between proportion of rural population in a district with childhood stunting and underweight. While theoretically it was expected that the urbanization leads to improve good health of children but, in present study, relationship was otherwise. One possible reason could be that if districts having good access of drinking water and sanitation facility in rural area, then rural area be no longer treat like rural area but will behaves like an urban or developed area. Another reason may be that there may be lot of other factors in the rural areas which might influence undernutrition but were not captured in this analysis. Maybe there is less poverty, high education in rural area or parents spend more time on child care etc. A lot of unexplained and omitted factors might be the reason for this negative association between undernutrition and percent rural population.

Malnutrition is more common among those districts where average birth order is higher and birth size of the baby is lower. This may be primarily due to unequal attention and nutritional shortfalls which increased division of available resources in the districts. So, study suggest that the lowering the birth order will be helpful in reducing the prevalence of malnutrition among under-five children in districts of India. However, it is quite encouraging to note that there were no significant sex differentials were noted in stunting and underweight, because now a day’s society is aware about the gender norms, equal food allocation and care practices for children.

The proportion of undernourished children is less among those districts where proportion of Muslim population is higher. This may be due to the consumption of food intake. Moreover, districts having higher share of scheduled caste/tribe’s population have lower proportion of stunted but higher proportion of underweight children. It might be notable that the recent ‘Maoist insurgency^1^’ in India is occurring mainly in districts where members of the scheduled caste/tribe communities predominantly live [35].

Malnutrition is a nation-wide phenomenon in India, as far as regional effects are concerned, malnutrition is concentrated in the least developed districts of central, east and northeast region, however more developed districts of west and southern region. Several studies found the definite assocaition between poverty and undernutrition at national and sub-natioanl level in India. Present study also found that the districts comes from Uttar Pradesh, Madhya Pradesh, Chattisgarh, Bihar, Jharkhand, Orisa and West Bengal are more likely to suffer from underweight. This may be because these states are facing high level of poverty and low level of development (EAG state). Due to underperformance of several demogrphic features, due to poor infrastruture this two regions is suffering from extent of childhood undernourshiment. On the other end, the distrcicts belongs to northeastern and southern regions are performing well in terms of both stunting and underweight compare to control region i.e., north region. Nevertheless, study further found that the distrcits comes from the west region showing higher proportion of stunted and underweight children in India. This higher extent of undernourished cases in the developed states of Gujarat and Maharashtra highlights that economic development fails to ensure better nutritional outcomes unless effectively backed by improvements in social and human development as well as institutional coverage.

## Conclusions

Using the data from fourth round of NFHS present study examined the socioeconomic inequalities in childhood malnutrition at district level in India. The findings from this study confirms the existence of socio-economic inequality in undernutrition in India. Findings of this study suggest that childhood malnutrition was mainly concentrate among the districts of central, eastern and western regions of the country. Decomposition approach found that practice of open defecation and birth order of child are positively influenced the socio-economic inequality in stunting and underweight. Further, socio-economic inequality in undernutrition is accelerated by height and education of mother and availability of safe drinking water in a district.. Present study concluded that the districts, that lied out in a spectrum of developmental diversity are required some specific set of information’s that covering socio-economic, demographic and health related quality of life of people in those backward districts. More generally, policies to avail improved water and sanitation facility to public and female literacy should be continued. It is also important to see that the benefits of both infrastructure and more general economic development are spread more evenly across districts.

## Data Availability

The data can be downloaded from the website of the Demographic and Health Survey (DHS) (https://dhsprogram.com/data/). The data for the current study were downloaded from the afore-mentioned website after taking permission.

## Abbreviations

EAG: Empowered action group
NFHS: National Family Health Survey
RBD: Regression based decomposition
SES: Socio-economic status
UN: United Nations
UNICEF: United Nations Children’s Fund

## Footnotes

1 The Maoist insurgency is an ongoing conflict between Maoist groups known as Naxalites or Naxals, and the Indian government. This is prevailing in the central part of the India (MP, Maharashtra, Bihar, Chhattisgarh, Jharkhand, Andhra Pradesh, Telangana, Orissa).
